# Supplementary material for: A meet-up of two second messengers: the c-di-AMP receptor DarB controls (p)ppGpp synthesis in Bacillus subtilis
Source: Nat Commun. 2021 Feb 22;12:1210. doi: 10.1038/s41467-021-21306-0 (PMC7900238; doi:10.1038/s41467-021-21306-0)
Supplement: Supplementary file 1 — Supplementary Information [file 41467_2021_21306_MOESM1_ESM.pdf]

SUPPLEMENTARY INFORMATION

A meet-up of two second messengers: The c-di-AMP receptor DarB controls (p)ppGpp synthesis in *Bacillus subtilis*

Larissa Krüger[1], Christina Herzberg[1], Dennis Wicke[1], Heike Bähre[2], Jana L. Heidemann[3], Achim Dickmanns[3], Kerstin Schmitt[4], Ralf Ficner[3] and Jörg Stülke[1]*

[1] Department of General Microbiology, Institute for Microbiology & Genetics, GZMB, Georg-August-University Göttingen, 37077 Göttingen, Germany

[2] Research Core Unit Metabolomics, Hannover Medical School, 30625 Hannover, Germany

[3] Department of Molecular Structural Biology, Institute for Microbiology & Genetics, GZMB, Georg-August-University Göttingen, 37077 Göttingen, Germany

[4] Department of Molecular Microbiology and Genetics, Service Unit LCMS Protein Analytics, Institute for Microbiology & Genetics, GZMB, Georg-August-University Göttingen, 37077 Göttingen, Germany

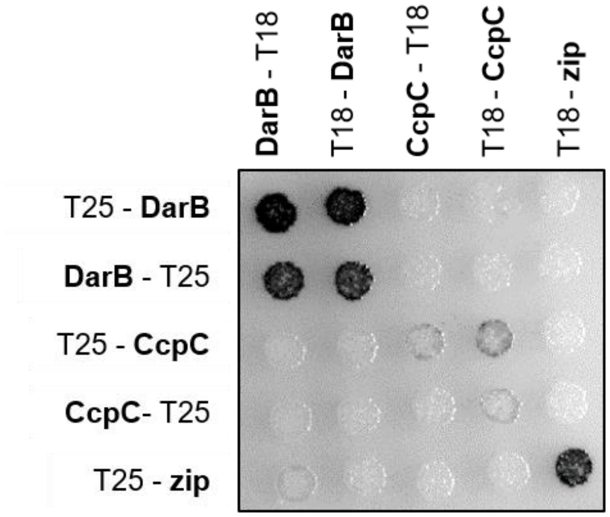

Supplementary Fig. 1. DarB does not interact with CcpC. Bacterial two-hybrid (BACTH) experiment testing for the interaction of DarB with CcpC. N- and C-terminal fusions of DarB and the CcpC variants to the T18 or T25 domain of the adenylate cyclase (CyaA) were created and the proteins were tested for interaction in *E. coli* BTH101. Dark colonies indicate an interaction that results in adenylate cyclase activity and subsequent expression of the reporter β-galactosidase. While DarB exhibits self-interaction, no interaction between DarB and CcpC could be detected. The experiment was conducted three times and a representative plate is shown.

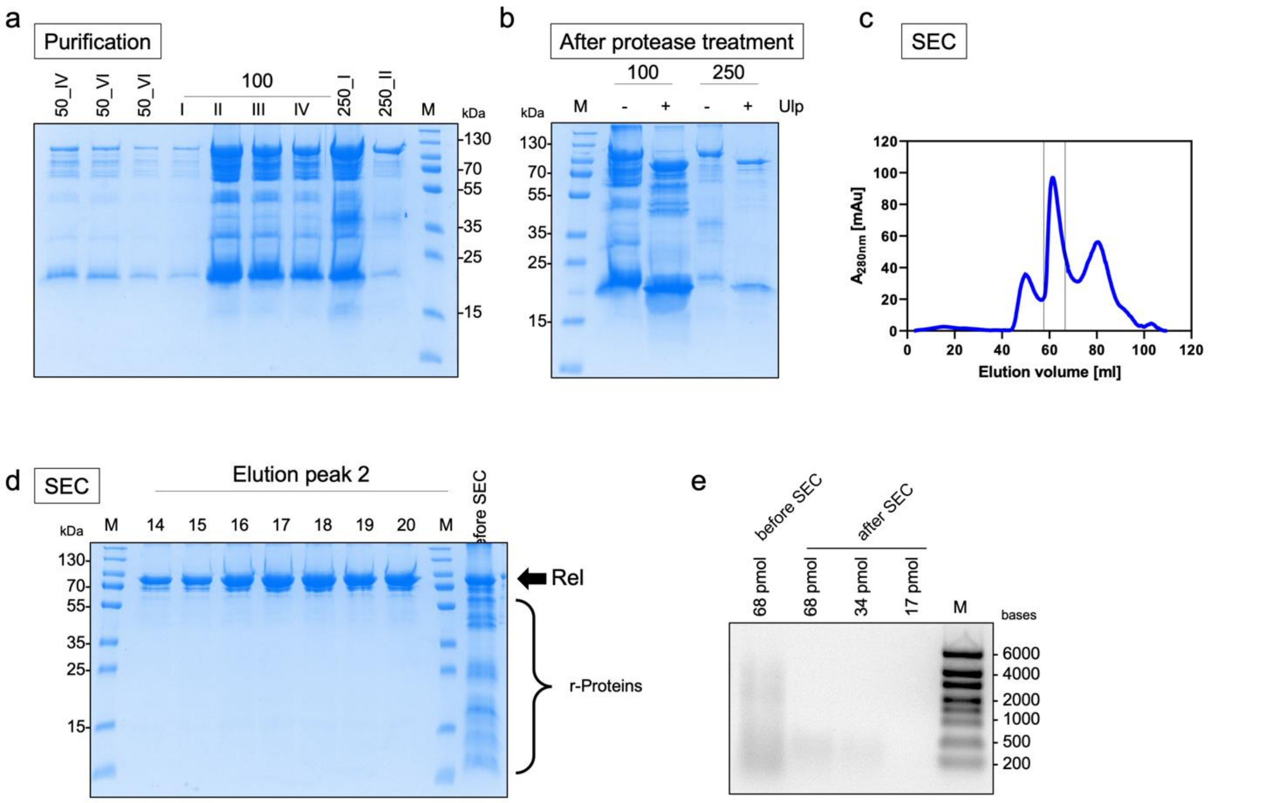

Supplementary Fig. 2. Purification of Rel. a, 10xHis-SUMO-tagged Rel (pVHP186) was overexpressed in *E. coli* Rosetta DE3 and purified in 750 mM KCl, 5 mM $MgCl_2$, 40 µM $MnCl_2$, 40 µM $Zn(OAc)_2$, 20 mM imidazole, 10% glycerol, 4 mM β-mercaptoethanol, 25 mM HEPES:KOH pH 8 via a Ni^{2+}nitrilotriacetic acid column. The protein was eluted with 100 and 250 mM imidazole and the elution fractions were analyzed by SDS-PAGE and Coomassie staining. **b**, The elution fractions were pooled and the 10xHis-SUMO-tag was cut off by overnight incubation with the SUMO protease. **c**, Size exlusion chromatography (SEC) was performed to remove contamination of the protein preparation. Source data are provided as a Source data file. The fractions that were used for the further experiments are highlighted and were analyzed by SDS-PAGE (**d**) and on a denaturing agarose gel to verify the absence of potentially contaminating RNA (**e**). The ratio between A_{260}/A_{280} was 0.9 indicating that the protein was almost free of contaminating RNA. Rel has been purified more than three times and representative gels are shown.

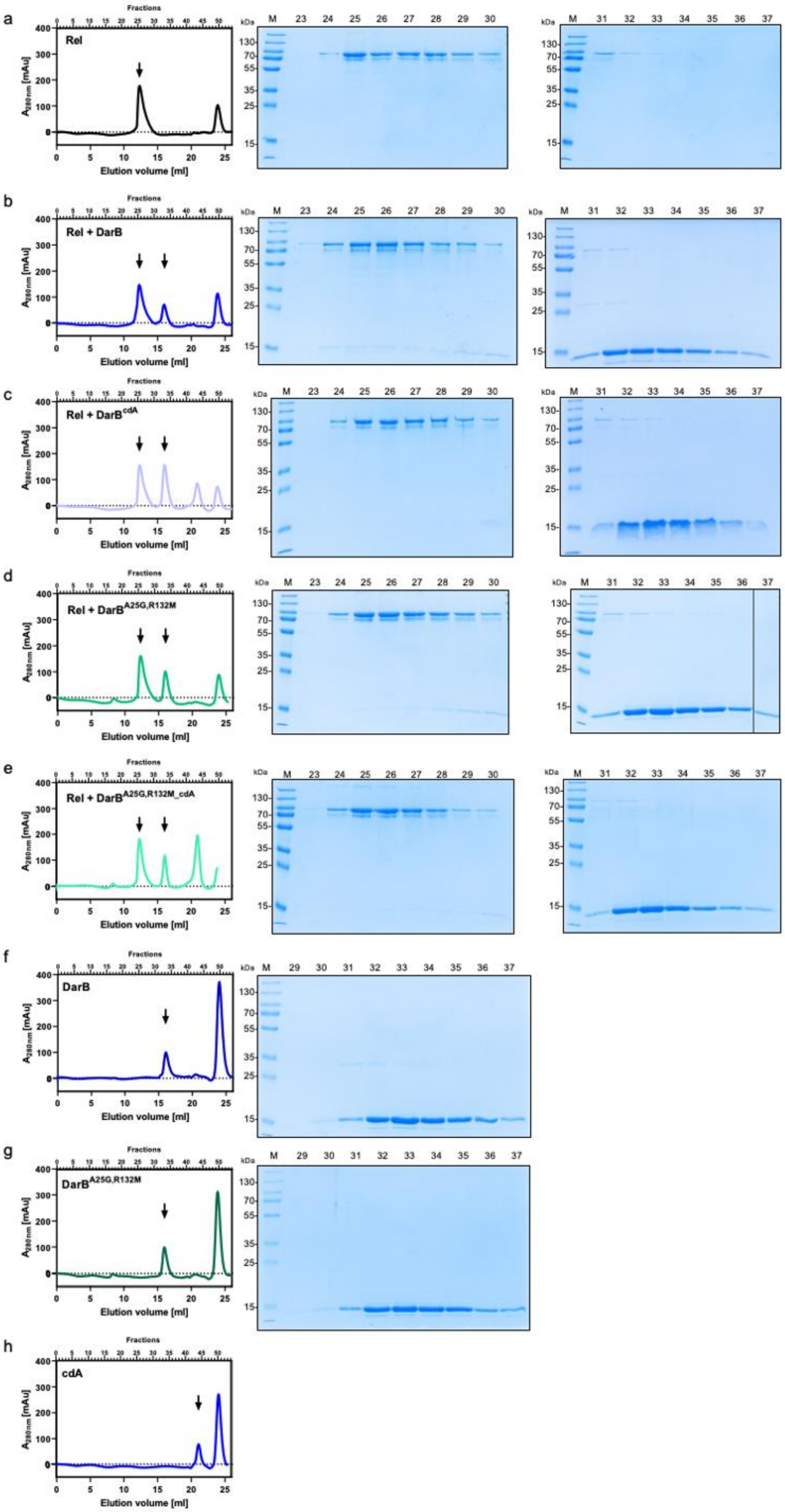
a
Rel
b
Rel + DarB
c
Rel + DarB^{cdA}
d
Rel + DarB^{A25G,R132M}
e
Rel + DarB^{A25G,R132M_cdA}
f
DarB
g
DarB^{A25G,R132M}
h
cdA
Fractions
A_{280nm} [mAu]
Elution volume [ml]
kDa
M
130
70
55
35
25
15

Supplementary Fig. 3. Analysis of the DarB-Rel-complex by size exclusion chromatography (SEC). Chromatograms of the SEC run are shown together with the SDS-gels of the relevant elution fractions (indicated above chromatogram and gel). Rel and DarB were used in equimolar concentrations. SEC runs of Rel alone (a) with DarB (b), DarB^{cdA} (c), DarB^{A25G,R132M} (d), or DarB^{A25G,R132M_cdA} (e), and as controls DarB (f), DarB^{A25G,R132M} (g), and c-di-AMP (h). Source data are provided as a Source data file. Abbreviation: cdA, c-di-AMP.

a

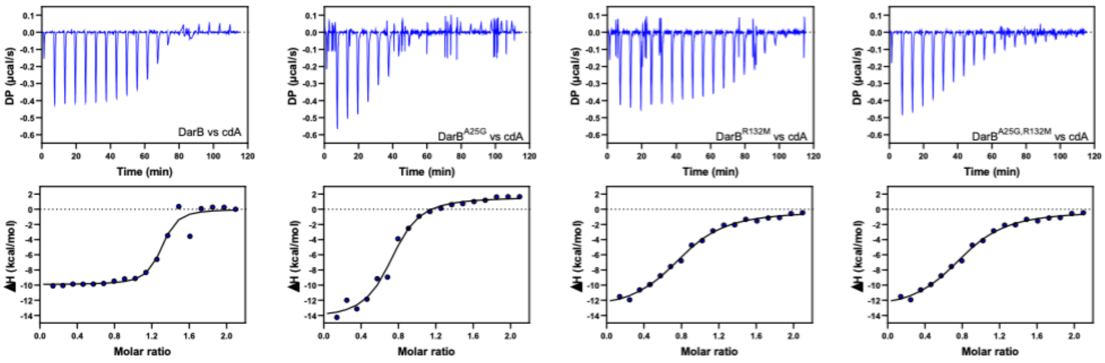

b

	K_D [M] cdA	N (sites)
**DarB**	$45.5x10^{-9} \pm 5.67x10^{-9}$	$1.26 \pm 5.3\ 10^{-3}$
**DarB^{A25G}**	$326x10^{-9} \pm 98.1x10^{-9}$	$0.713 \pm 2.1x10^{-2}$
**DarB^{R132M}**	$292x10^{-9} \pm 110x10^{-9}$	1.82 ± 0.115
**DarB^{A25G,R132M}**	$880x10^{-9} \pm 175x10^{-9}$	$0.804 \pm 2.0x10^{-2}$

c

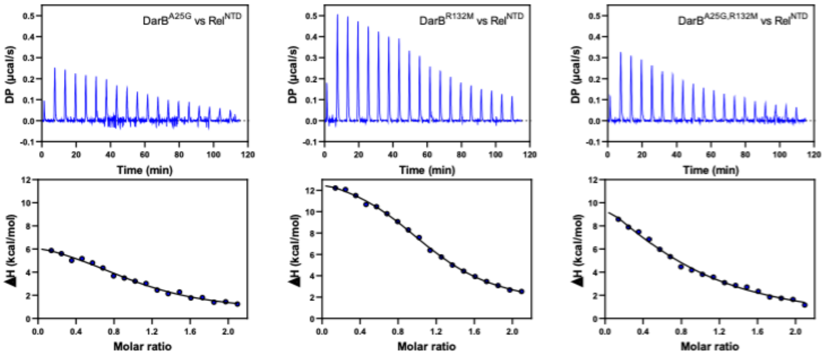

Supplementary Fig. 4. Analysis of the binding affinities of DarB, and the DarB mutants towards c-di-AMP and Rel^{NTD}. a, The ability of the DarB wild type and the mutant proteins to bind c-di-AMP was assessed by Isothermal titration calorimetry (ITC). The cell and the syringe contained 10 µM DarB and 100 µM c-di-AMP, respectively. Titration profiles and the determined molar ratios. **b**, Calculated K_D values for binding of c-di-AMP, as well as the determined number of binding ligand sites. **c**, The interaction of the DarB mutant proteins with Rel^{NTD} was investigated with ITC. The cell and the syringe contained 10 µM Rel and 100 µM DarB, respectively. Source data are provided as a Source data file. Abbreviation: cdA, c-di-AMP.

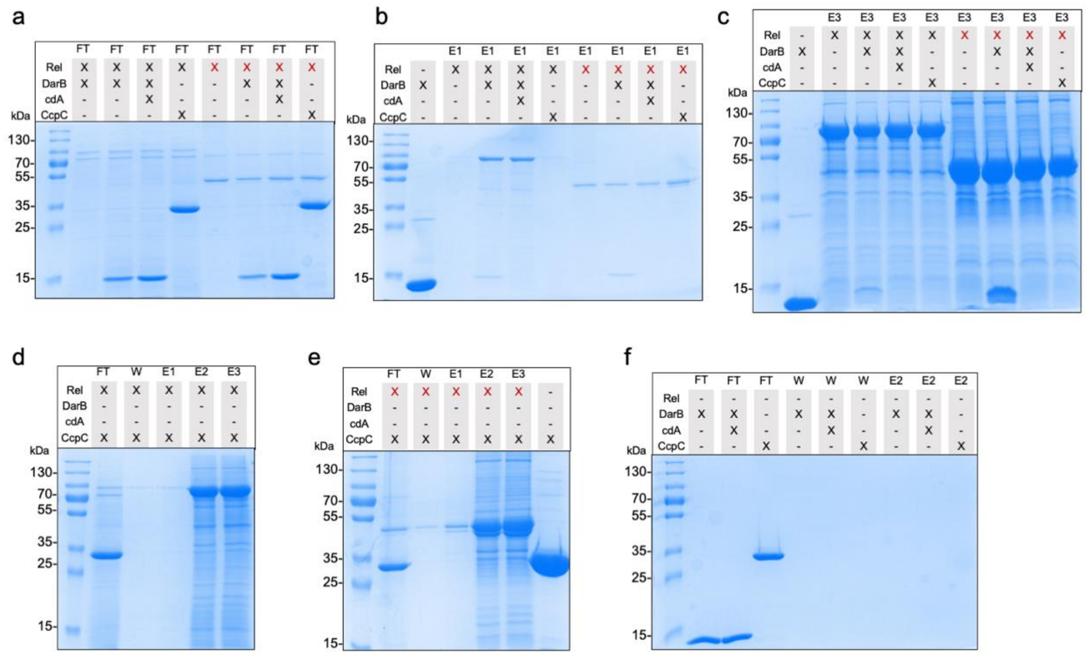

Supplementary Fig. 5. *In vitro* interaction experiment between DarB and Strep-Rel or Strep-Rel^{NTD}. Strep-Rel or Strep-Rel^{NTD} were immobilized onto a StrepTactin column and incubated with DarB, DarB preincubated with c-di-AMP, or the control protein CcpC. The eluates were analyzed by SDS-PAGE. Control gels are shown, the presence of the N-terminal Strep- Rel^{NTD} variant is indicated by a red cross. **a**, Flow through (FT) after loading of DarB or CcpC; **b**, Elution fractions 1; **c**, Elution fractions 3; **d**, SDS PAGE showing all fractions of the negative control CcpC and Strep-Rel; **e**, SDS PAGE showing all fractions of the negative control CcpC and Strep- Rel^{NTD}; **f**, SDS PAGE with all fractions of DarB/ CcpC to exclude unspecific binding to the column. Abbreviation: cdA, c-di-AMP. The experiment was conducted three times and a representative gel is shown.

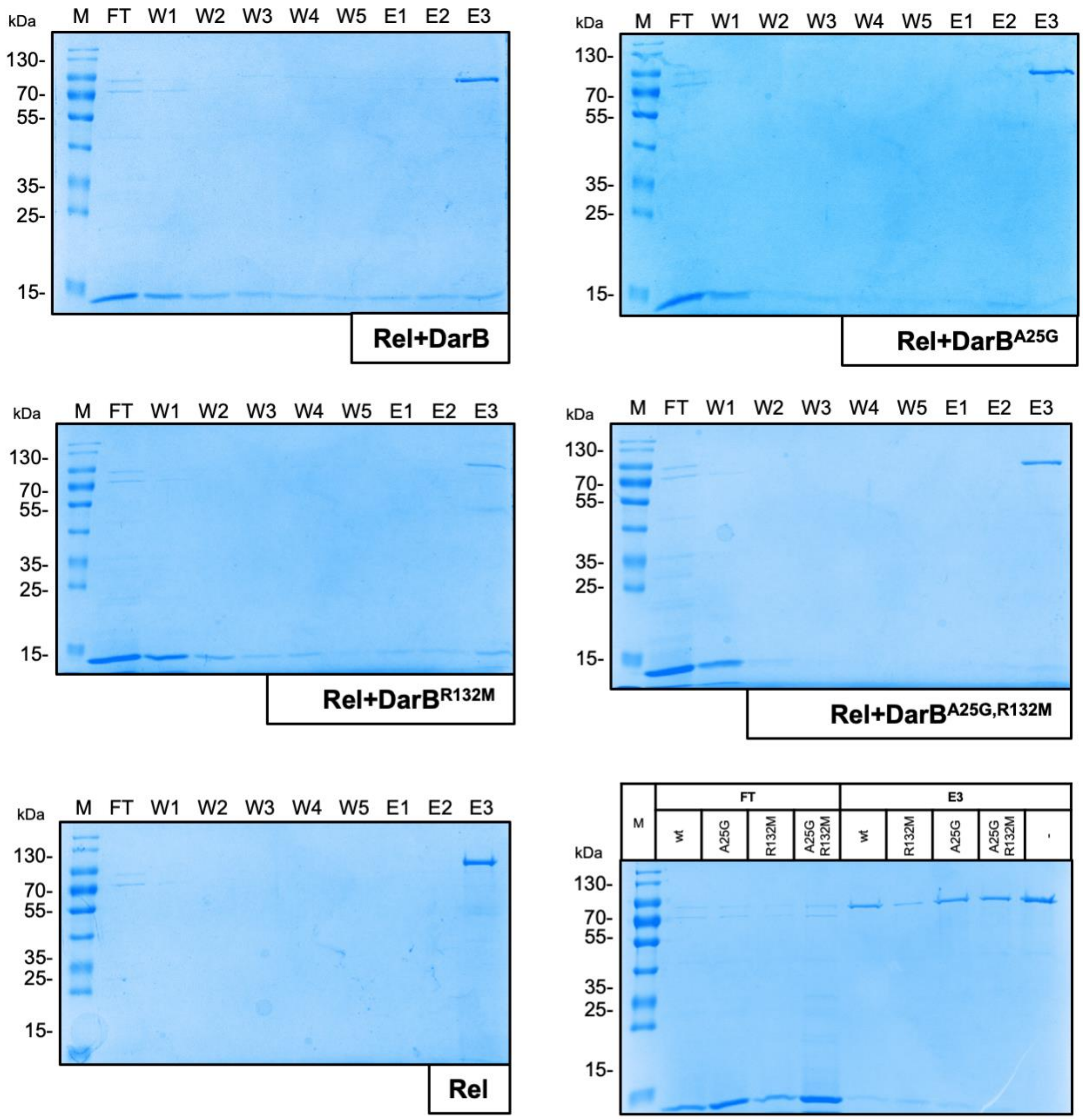

Supplementary Fig. 6. ***In vitro*** **interaction experiment between DarB, DarB^{A25G}, DarB^{R132M}, DarB^{A25G,R132M}, and Strep-Rel.** Strep-Rel was immobilized onto a StrepTactin column and incubated with DarB, or the DarB mutants. The flow through (FT), the wash fractions (W), and the eluates were analyzed by SDS-PAGE. The experiment was conducted three times and a representative gel is shown.

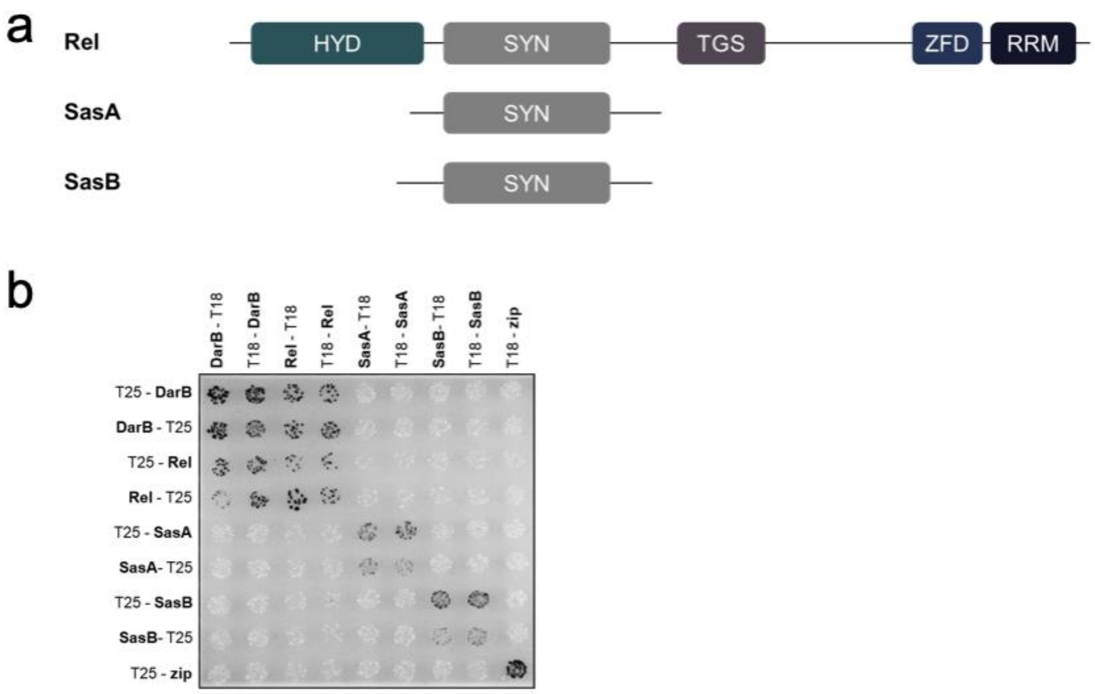

Supplementary Fig. 7. DarB does not interact with the small alarmone synthetases SasA and SasB. a, Domain organization of Rel and the small alarmone sythetases SasA and SasB. **b**, Bacterial two-hybrid (BACTH) experiment testing for the interaction of DarB with Rel, SasA and SasB. N- and C-terminal fusions of DarB and Rel, SasA or SasB to the T18 or T25 domain of the adenylate cyclase (CyaA) were created and the proteins were tested for interaction in *E. coli* BTH101. Dark colonies indicate an interaction that results in adenylate cyclase activity and subsequent expression of the reporter β-galactosidase. While DarB exhibits an interaction with Rel, no interaction between DarB and SasA or SasB could be detected. The experiment was conducted three times and a representative plate is shown.

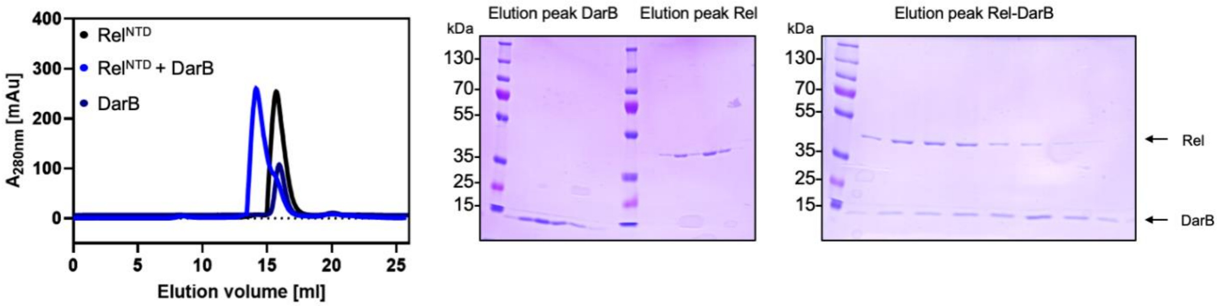

Supplementary Fig. 8. *In vitro* analysis of the DarB-Rel^{NTD} complex. Size-exclusion chromatography and multi-angle light scattering (SEC-MALS) of DarB (dark blue), Rel (black), and the DarB-Rel^{NTD}-complex (blue). Coomassie-stained gels of the relevant elution fractions are depicted next to the chromatogram. Source data are provided as a Source data file.

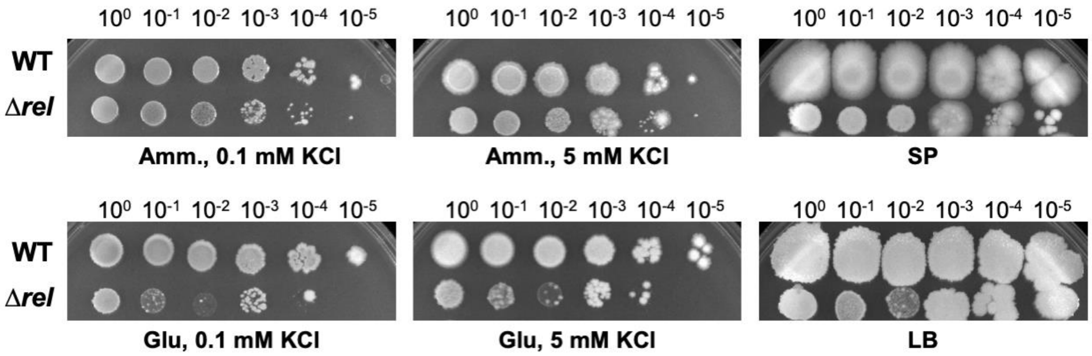

Supplementary Fig. 9. Phenotypes of a *B. subtilis rel* mutant. Growth assay of *B. subtilis* wild type, and GP3419 (Δ*rel*). The *B. subtilis* strains were cultivated in MSSM minimal medium with 0.1 mM KCl and ammonium. The cells were harvested, washed, and the OD_{600} was adjusted to 1.0. Serial dilutions were dropped onto MSSM minimal plates with the indicated potassium concentration and ammonium or glutamate, or on sporulation (SP) and LB complex medium plates. The growth experiment was conducted three times and representative plates are shown.

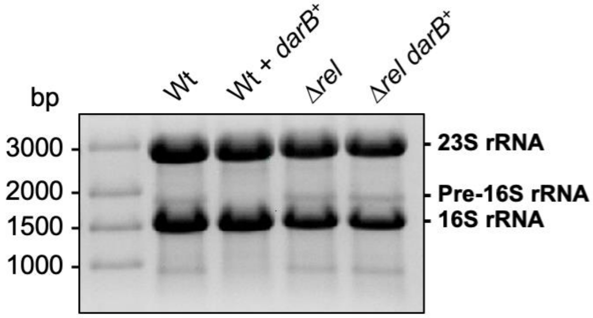

Supplementary Fig. 10. Analysis of rRNA profiles in *B. subtilis* mutants. Cultures were grown in minimal medium with 0.1 mM KCl. Total RNA was extracted and 3 µg were resolved on a 1% agarose formaldehyde gel and stained with ethidium bromide. Positions of the ribosomal rRNAs are indicated. The experiment was conducted three times and a representative RNA gel is shown.

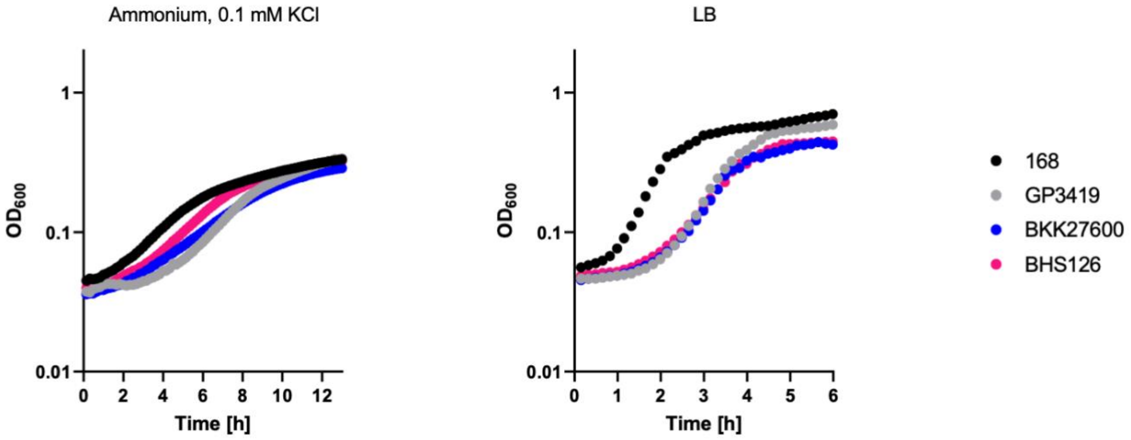

Supplementary Fig. 11. Phenotypes of a *B. subtilis rel* mutants. Growth experiments of the *B. subtilis* wild type strain 168 and the isogenic Δ*rel* mutants GP3419, BHS126, and BKK27600 in MSSM minimal medium with 0.1 mM KCl (left) or in complex medium (lysogeny broth) (right). Growth was monitored in an Epoch 2 Microplate Spectrophotometer (BioTek Instruments) at 37°C with linear shaking at 237 cpm (4 mm) for 12 h. The growth experiment was conducted three times and representative curves from one experiment are shown. Source data are provided as a Source data file.

Full scan Figure 1c

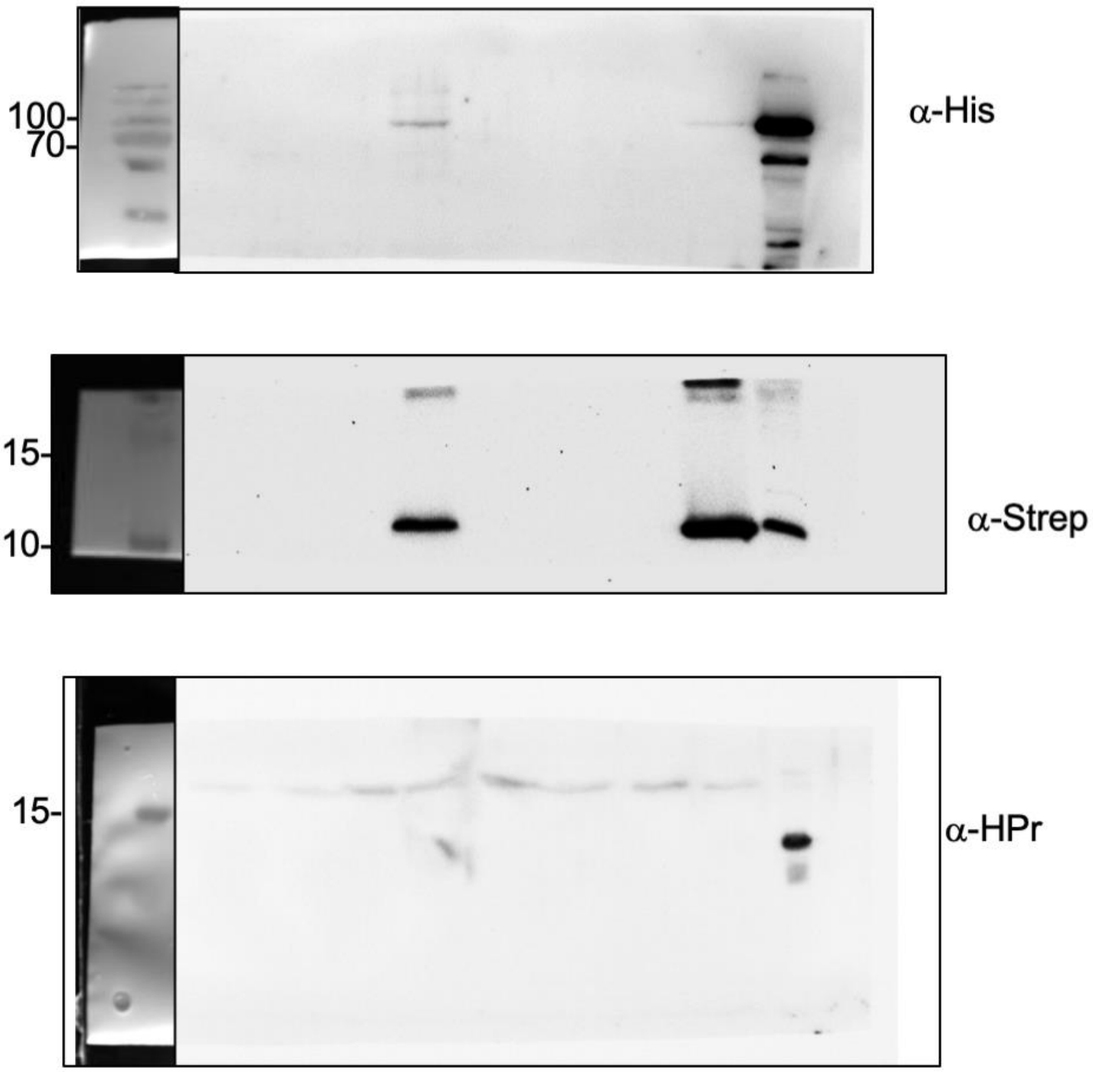

Supplementary Fig. 12. Uncropped images for Figure 1c.

Supplementary Table 1

Analysis of excised gel bands from the *in vitro* pulldown experiment with Strep-Rel

Peptides identified by search of MS/MS2 data against *B. subtilis* specific protein database (UniProt Proteome ID UP000001570)

List of the samples

LK29 Excised gel band 1 (Rel, DarB)

LK30 Excised gel band 2 (Rel, DarB, c-di-AMP)

LK31 Excised gel band 3 (Rel^{NTD}, DarB)

LK32 Excised gel band 4 (Rel^{NTD}), DarB, c-di-AMP)

**LK29**				
**Accession**	**Description**	**Coverage (%)**	**Number of identified peptides**	**Number of peptide sequence matches**
O31698	DarB	89	8	576
O54408	Rel	24	14	140

**LK30**				
**Accession**	**Description**	**Coverage (%)**	**Number of identified peptides**	**Number of peptide sequence matches**
O54408	Rel	41	30	234
O31698	DarB	89	8	164
Q06796	RplK	6	1	2

P12877	RplE	7	1	2

LK31				
**Accession**	**Description**	**Coverage (%)**	**Number of identified peptides**	**Number of peptide sequence matches**
O31698	DarB	89	11	1659
O54408	Rel	24	13	193
Q06796	RplK	6	1	3

LK32				
**Accession**	**Description**	**Coverage (%)**	**Number of identified peptides**	**Number of peptide sequence matches**
O31698	DarB	89	8	212
O54408	Rel	29	20	118
P18156	GlpF	10	1	1
P54469	YqfD	2	1	1

Supplementary Table 2

Plasmids used in this study

**Name**	**Vector**	**Insert**	**Reference**
pGP635	pGP380/ XbaI + PstI	PCR-Product *darB*, TK01/TK02 (XbaI + PstI)	This study
pGP706	pWH844/ SalI	PCR-Prod. *ccpC,* HMB9/HMB11/ (SalI)	1
pGP767	pGP382/ XbaI + PstI	PCR-Prod. *darB*, TK03/TK04 (XbaI + PstI)	This study
pGP2972	pET-SUMO/ BsaI + XhoI	PCR-Prod. *darB,* LK80/LK81 (BsaI + XhoI)	This study
pGP2974	pUT18/ XbaI + KpnI	PCR- Prod. *darB,* LK130/LK131 (XbaI + KpnI)	This study
pGP2975	pUT18c/ XbaI + KpnI	PCR- Prod. *darB*, LK130/LK131 (XbaI + KpnI)	This study
pGP2976	pKT25/ XbaI + KpnI	PCR- Prod. *darB*, LK130/LK131 *(XbaI + KpnI)*	This study
pGP2977	p25-N/ XbaI + KpnI	PCR- Prod. *darB*, LK130/LK131 (XbaI + KpnI)	This study
pGP2982	pUT18/ XbaI + KpnI	PCR- Prod. *ccpC,* LK161/LK162 *(XbaI + KpnI)*	This study
pGP2983	pUT18c/ XbaI + KpnI	PCR- Prod. *ccpC,* LK161/LK162 (XbaI + KpnI)	This study
pGP2984	pKT25/ XbaI + KpnI	PCR- Prod. *ccpC,* LK161/LK162 (XbaI + KpnI)	This study
pGP2985	p25-N/ XbaI + KpnI	PCR- Prod. *ccpC,* LK161/LK162 (XbaI + KpnI)	This study
pGP3306	pBQ200/ XbaI + PstI	PCR-Prod. *darB,* LK209/TK02 (XbaI + PstI)	This study
pGP3330	pWH844/ BamHI + SalI	PCR-Prod. *rel,* LK283/LK284 (BamHI + SalI)	This study
pGP3336	pUT18/ XbaI + KpnI	PCR- Prod. *sasA,* LK312/LK313 (XbaI + KpnI)	This study
pGP3337	pUT18c/ XbaI + KpnI	PCR- Prod. *sasA,* LK312/LK313 (XbaI + KpnI)	This study
pGP3338	pKT25/ XbaI + KpnI	PCR- Prod. *sasA,* LK312/LK313 (XbaI + KpnI)	This study
pGP3339	p25-N/ XbaI + KpnI	PCR- Prod. *sasA,* LK312/LK313 (XbaI + KpnI)	This study
pGP3344	pUT18/ XbaI + KpnI	PCR- Prod. *rel,* LK287/LK288 (XbaI + KpnI)	This study
pGP3345	pUT18c/ XbaI + KpnI	PCR- Prod. *rel,* LK287/LK288 (XbaI + KpnI)	This study

pGP3346	pKT25/ XbaI + KpnI	PCR- Prod. *rel,* LK287/LK288 (XbaI + KpnI)	This study
pGP3347	p25-N/ XbaI + KpnI	PCR- Prod. *rel,* LK287/LK288 (XbaI + KpnI)	This study
pGP3348	pGP172/ KpnI + BamHI	PCR-Prod. *rel* LK310/LK311 (KpnI + BamHI)	This study
pGP3350	pGP172/ KpnI + BamHI	PCR-Produkt rel^{NTD} LK310/LK333 (KpnI + BamHI)	This study
pGP3411	pUT18/ XbaI + KpnI	PCR- Prod. *sasB,* LK314/LK315 (XbaI + KpnI)	This study
pGP3412	pUT18c/ XbaI + KpnI	PCR- Prod. *sasB,* LK314/LK315 (XbaI + KpnI)	This study
pGP3413	pKT25/ XbaI + KpnI	PCR- Prod. *sasB,* LK314/LK315 (XbaI + KpnI)	This study
pGP3414	p25-N/ XbaI + KpnI	PCR- Prod. *sasB,* LK314/LK315 (XbaI + KpnI)	This study
pGP3415	pUT18/ XbaI + KpnI	PCR- Prod. $rel^{SYN-RRM}$, LK316/LK288 (XbaI + KpnI)	This study
pGP3416	pUT18c/ XbaI + KpnI	PCR- Prod. $rel^{SYN-RRM}$, LK316/LK288 (XbaI + KpnI)	This study
pGP3417	pKT25/ XbaI + KpnI	PCR- Prod. $rel^{SYN-RRM}$, LK316/LK288 (XbaI + KpnI)	This study
pGP3418	p25-N/XbaI + KpnI	PCR- Prod. $rel^{SYN-RRM}$, LK316/LK288 (XbaI + KpnI)	This study
pGP3419	pUT18/ XbaI + KpnI	PCR- Prod. rel^{NTD}, LK287/LK317 (XbaI + KpnI)	This study
pGP3420	pUT18c/ XbaI + KpnI	PCR- Prod. rel^{NTD}, LK287/LK317 (XbaI + KpnI)	This study
pGP3421	pKT25/ XbaI + KpnI	PCR- Prod. rel^{NTD}, LK287/LK317 (XbaI + KpnI)	This study
pGP3422	p25-N/ XbaI + KpnI	PCR- Prod. rel^{NTD}, LK287/LK317 (XbaI + KpnI)	This study
pGP3429	pWH844/ BamHI + SalI	PCR-Prod. rel^{NTD}, LK283 + LK358 (BamHI + SalI)	This study
pGP3437	pBQ200/ XbaI + PstI	PCR-Prod. $darB^{A25G}$, LK209+ TK02 + LK372 (XbaI + PstI)	This study
pGP3441	pBQ200/ XbaI + PstI	PCR-Prod. $darB^{R132M}$, LK209+ TK02 + LK376 (XbaI + PstI)	This study
pGP3444	pET-SUMO/ XhoI + BsaI	PCR-Prod. $darB^{A25G}$, LK80 + LK81 (XhoI + BsaI)	This study

pGP3448	pET-SUMO/XhoI+BsaI	PCR-Prod. *darB*^{R132M}, LK80 + LK81 (XhoI + BsaI)	This study
pGP3460	pET-SUMO/XhoI+BsaI	PCR-Prod. *darB*$^{A25G/R132M}$, LK80 + LK81 (XhoI + BsaI)	This study
pGP3601	pBQ200/XbaI+PstI	PCR-Prod. *darB*^{A25G,R132M}, LK209+ TK02 + LK376 (XbaI + PstI)	This study
pVHP186	pET24d	*rel*	2

Supplementary Table 3

Oligonucleotides used in this study

Underlined: restriction sites; bold: introduction of additional bases or mutations; fwd: forward primer; rev: reverse primer; RBS: ribosomal binding site.

**Name**	**Sequence**	**Gene/purpose**	**Remarks**
LK80	AAAGGTCTCATGGTATGATAAGCTTACAATCAGATCAACTTCTTGAGG	fw, *darB,* N-terminal 6xHis-tag fusion in pET-SUMOadapt	*Bsa*I; ATGGT extra for proper cloning
LK81	TTTCTCGAGCTACTTATTCAATGAGCGTATATGCTTATTCAATTCC	rev, *darB,* N-terminal 6xHis-tag fusion in pET-SUMOadapt	*Xho*I
LK130	AAATCTAGAGATGATAAGCTTACAATCAGATCAACTTCTTGAGG	fwd, *darB* bacterial two hybrid, *Xba*I	Extra base to restore reading frame, *Xba*I
LK131	TTTGGTACCCGCTTATTCAATGAGCGTATATGCTTATTCAATTCC	rev, *darB* bacterial two hybrid, *Kpn*I	Two extra bases to restore reading frame, *Kpn*I
LK161	AAATCTAGAGATGCAGCTTCAAGAGCTTCATATGCTCG	fwd, *ccpC* bacterial two hybrid, *Xba*I	Extra base to restore reading frame, *Xba*I
LK162	TTTGGTACCGCAAATGGATTTTCCTGATCCAGCATATCCTTTATCA	rev, *ccpC* bacterial two hybrid, *Kpn*I	Two extra bases to restore reading frame, *Kpn*I
LK209	AAATCTAGAATTAAAGAGGAGAAATTAACTATGATAAGCTTACAATCAGATCAACTTCTTGAGG	fw, *darB,* pBQ200	XbaI, + RBS
LK283	AAAGGATCCATGGCGAACGAACAAGTATTGACT	fw, *rel,* pWH844	BamHI
LK284	TTTGTCGACTTAGTTCATGACGCGGCGCA	rev, *rel,* pWH844	SalI

LK287	AAA<u>TCTAGA</u>GATGGCGAACGAACAAGTATTGACT	fwd, *rel* bacterial two hybrid, *Xba*I	*Extra base* to restore reading frame, *Xba*I
LK288	TTT<u>GGTACC</u>GCGTTCATGACGCGGCGCA	rev, *rel* bacterial two hybrid, *Kpn*I	Two extra bases to restore reading frame, *Kpn*I
LK310	AAA<u>GGTACC</u>AATGGCGAACGAACAAGTATTGACT	fw, *rel,* N-terminal Strep-tag fusion in pGP172	Extra base to restore reading frame, *Kpn*I
LK311	TTT<u>GGATCC</u>TTAGTTCATGACGCGGCGCA	rev, *rel,* N-terminal Strep-tag fusion in pGP172	*BamH*I
LK312	AAA<u>TCTAGA</u>GATGGATTTATCTGTAACACATATGGAC	fwd, *sasA* bacterial two hybrid, *Xba*I	Extra base to restore reading frame, *Xba*I
LK313	TTT<u>GGTACC</u>GCATCCACTTCTTTCTTAATCCCCAG	rev, *sasA* bacterial two hybrid, *Kpn*I	Two extra bases to restore reading frame, *Kpn*I
LK314	AAA<u>TCTAGA</u>GATGGATGACAAACAATGGGAGC	fwd, *sasB* bacterial two hybrid, *Xba*I	Extra base to restore reading frame, *Xba*I
LK315	TTT<u>GGTACC</u>GCTTGTTGCTCGCTTCCTTTTTTCTTTC	rev, *sasB* bacterial two hybrid, *Kpn*I	Two extra bases to restore reading frame, *Kpn*I
LK316	AAA<u>TCTAGA</u>GCTGGAAATTTTTGCTCCTTTGGC	fwd, $rel^{SYN\text{-}RRM}$, bacterial two hybrid/ pGP888, *Xba*I	Extra base to restore reading frame, *Xba*I
LK317	TTT<u>GGTACC</u>GCAGAGAACAAATCAATTTTGAGCGATTC	rev, rel^{NTD}, bacterial two hybrid, *KpnI*	Two extra bases to restore reading frame, *Kpn*I
LK333	TTT<u>GGATCC</u>TTATTAAGAGAACAAATCAATTTTGAGCGATTC	rev, rel^{NTD}, N-terminal Strep-tag fusion in pGP172, BamHI	BamHI + 2xSTOPP
LK358	TTT<u>GTCGAC</u>TCATCAAGAGAACAAATCAATTTTGAGCGATTC	rev, rel^{NTD}, N-terminal Strep-tag fusion in pWH844	STOPP + SalI

LK372	[Phos]GCGGACAAAGTAGGGCACGTGCAAGT	CCR-Primer *darB* (A25G)	5´-P
LK376	[Phos]ACAAAAACCGGATACGTCGCCATCCC	CCR-Primer *darB* (T46V)	5´-P
TK01	AAATCTAGAATGATAAGCTTACAATCAGATCAACTTCTTG	fwd, *darB*, N-terminal Strep-tag fusion in pGP380	*XbaI*
TK02	TTTCTGCAGCTACTTATTCAATGAGCGTATATGCTTATTCA	rev, *darB*, N-terminal Strep-tag fusion in pGP380	*PstI*

References

1. Blencke, H. M. et al. Regulation of *citB* expression in *Bacillus subtilis*: Integration of multiple metabolic signals in the citrate pool and by the general nitrogen regulatory system. *Arch. Microbiol*. **185**, 136-146 (2006).
2. Takada, H., et al. The C-terminal RRM/ACT domain is crucial for fine-tuning the activation of “long” RelA-SpoT homolog enzymes by ribosomal complexes. *Front. Microbiol*. **11**, 277 (2020).
