# Supplementary material for: A meet-up of two second messengers: the c-di-AMP receptor DarB controls (p)ppGpp synthesis in Bacillus subtilis
Source: Nat Commun. 2021 Feb 22;12:1210. doi: 10.1038/s41467-021-21306-0 (PMC7900238; doi:10.1038/s41467-021-21306-0)
Supplement: Supplementary file 3 — Description of Additional Supplementary Files [file 41467_2021_21306_MOESM3_ESM.docx]

**Description of Additional Supplementary Files**

**File Name: Supplementary Data 1**

**Description: Analysis of eluates from the DarB pulldown experiment with *Bacillus subtilis* cell extract**

Peptides identified by search of MS/MS2 data against *B. subtilis* specific protein database (UniProt Proteome ID UP000001570)

**File Name: Supplementary Data 2**

**Description: Analysis of the *in vivo* interaction experiment of DarB-Strep with low (0.1 mM) and high (5 mM) potassium concentrations**

Peptides identified by search of MS/MS2 data against *B. subtilis* specific protein database (UniProt Proteome ID UP000001570)
